# Interactions Between Immunomodulatory Biomaterials and Immune Microenvironment: Cues for Immunomodulation Strategies in Tissue Repair

**DOI:** 10.3389/fbioe.2022.820940

**Published:** 2022-05-13

**Authors:** Yi Chen, Weiyan Sun, Hai Tang, Yingze Li, Chen Li, Long Wang, Jiafei Chen, Weikang Lin, Shenghui Li, Ziwen Fan, Yu Cheng, Chang Chen

**Affiliations:** ^1^ Department of Thoracic Surgery, Shanghai Pulmonary Hospital, Tongji University, Shanghai, China; ^2^ Institute for Regenerative Medicine, Institute for Translational Nanomedicine, Shanghai East Hospital, Tongji University School of Medicine, Shanghai, China; ^3^ School of Materials Science and Engineering, Tongji University, Shanghai, China; ^4^ The Institute for Biomedical Engineering and Nano Science, Tongji University School of Medicine, Shanghai, China

**Keywords:** immunomodulatory biomaterials, foreign body response, tissue repair, immune microenvironment, self-adaptive interaction

## Abstract

The foreign body response (FBR) caused by biomaterials can essentially be understood as the interaction between the immune microenvironment and biomaterials, which has severely impeded the application of biomaterials in tissue repair. This concrete interaction occurs via cells and bioactive substances, such as proteins and nucleic acids. These cellular and molecular interactions provide important cues for determining which element to incorporate into immunomodulatory biomaterials (IMBs), and IMBs can thus be endowed with the ability to modulate the FBR and repair damaged tissue. In terms of cellular, IMBs are modified to modulate functions of immune cells, such as macrophages and mast cells. In terms of bioactive substances, proteins and nucleic acids are delivered to influence the immune microenvironment. Meanwhile, IMBs are designed with high affinity for spatial targets and the ability to self-adapt over time, which allows for more efficient and intelligent tissue repair. Hence, IMB may achieve the perfect functional integration in the host, representing a breakthrough in tissue repair and regeneration medicine.

## 1 Introduction

With the development of tissue engineering and regenerative medicine, biomaterials have been explored to design implants targeting promoting wound healing (Kim et al., 2017; Castaño et al., 2018; Nourian Dehkordi et al., 2019; Oliva and Almquist, 2020; Sharifi et al., 2020), repairing injured tissue (Han et al., 2019; Gaharwar et al., 2020; Primavera et al., 2020), constructing bionic organs (Eke et al., 2017; Lee et al., 2017; Brennan et al., 2020; Lee et al., 2021; Wang et al., 2021), and so on (Chung et al., 2017; Pugliese and Gelain, 2017; Liu et al., 2018; Wu et al., 2018; Sultankulov et al., 2019). Some of these biomaterials such as wound healing adhesives and bone cement, have been applied in clinical situations and have benefited patients worldwide (Schmalz and Galler, 2017; Perez et al., 2018; Turnbull et al., 2018; Zhang et al., 2018; Kargozar et al., 2019; Cheng et al., 2020a; Armiento et al., 2020; Khare et al., 2020). Regenerative medicine approaches that repair damaged and malfunctioned tissues using biomaterials have a promising future (Li et al., 2020; Liu et al., 2021; Peng et al., 2020; Xu et al., 2020; Kumar et al., 2020; D'Este et al., 2018; Kowalski et al., 2018; Defraeye and Martynenko, 2018).

However, when biomaterials are integrated into the host, the foreign body response (FBR) inevitably arises (Doloff et al., 2017; Ibrahim et al., 2017; Chandorkar et al., 2018; Sharifi et al., 2019; Veiseh and Vegas, 2019), in which the immune microenvironment interacts with biomaterials *via* humoral and cellular factors, and this process determines the success of the integration and the biological performance of the biomaterials (Anderson et al., 2008; Sadtler et al., 2016a; Chu et al., 2019). When the FBR is excessively happened, inflammation, fibrosis, infection, and thrombosis can occur (Bitar, 2015; Adu-Berchie and Mooney, 2020), resulting in material degradation, fiber proliferation, and so on, which impedes the morphological and functional maintenance of biomaterials *in vivo* (Martin and Leibovich, 2005; Bitar, 2015).

Immunomodulatory biomaterials (IMBs) are defined as biomaterials with the design to control the FBR processes in order to biomaterial−tissue integration and tissue repair, which are a feasible principle of biomaterial development (Sadtler et al., 2016b; Chu et al., 2017a; Andorko and Jewell, 2017; Chu et al., 2017b; Dziki and Badylak, 2018; Lee et al., 2019; Wolf et al., 2019; Adu-Berchie and Mooney, 2020; Hu et al., 2021). In the design of the IMBs, the immune-related substances or cells can be attached to the biomaterials, to produce an immunomodulatory effect on the microenvironment and to control the FBR. Since the FBR essentially arises from the interaction between the immune microenvironment and the biomaterials, many researchers have designed IMBs based on modulating these interactions, and the design element used were coming from the analysis of specific interactions, such as those between cells and bioactive substances which including proteins, and nucleic acids, and so on (Dellacherie et al., 2019; Eslami-Kaliji et al., 2020; Lasola et al., 2020).

In this review, we summarized the development of IMBs incorporating the cells and substances involved in the interaction between IMBs and the immune microenvironment. The principle and aim of IMB modification should be the modulation of the FBR by regulating interactions, resulting in IMBs that can function in tissue regeneration and repair.

## 2 The Mechanism of the Foreign Body Response: The Interactions Between Immunomodulatory Biomaterial and Immune Microenvironment

**FIGURE 1:**
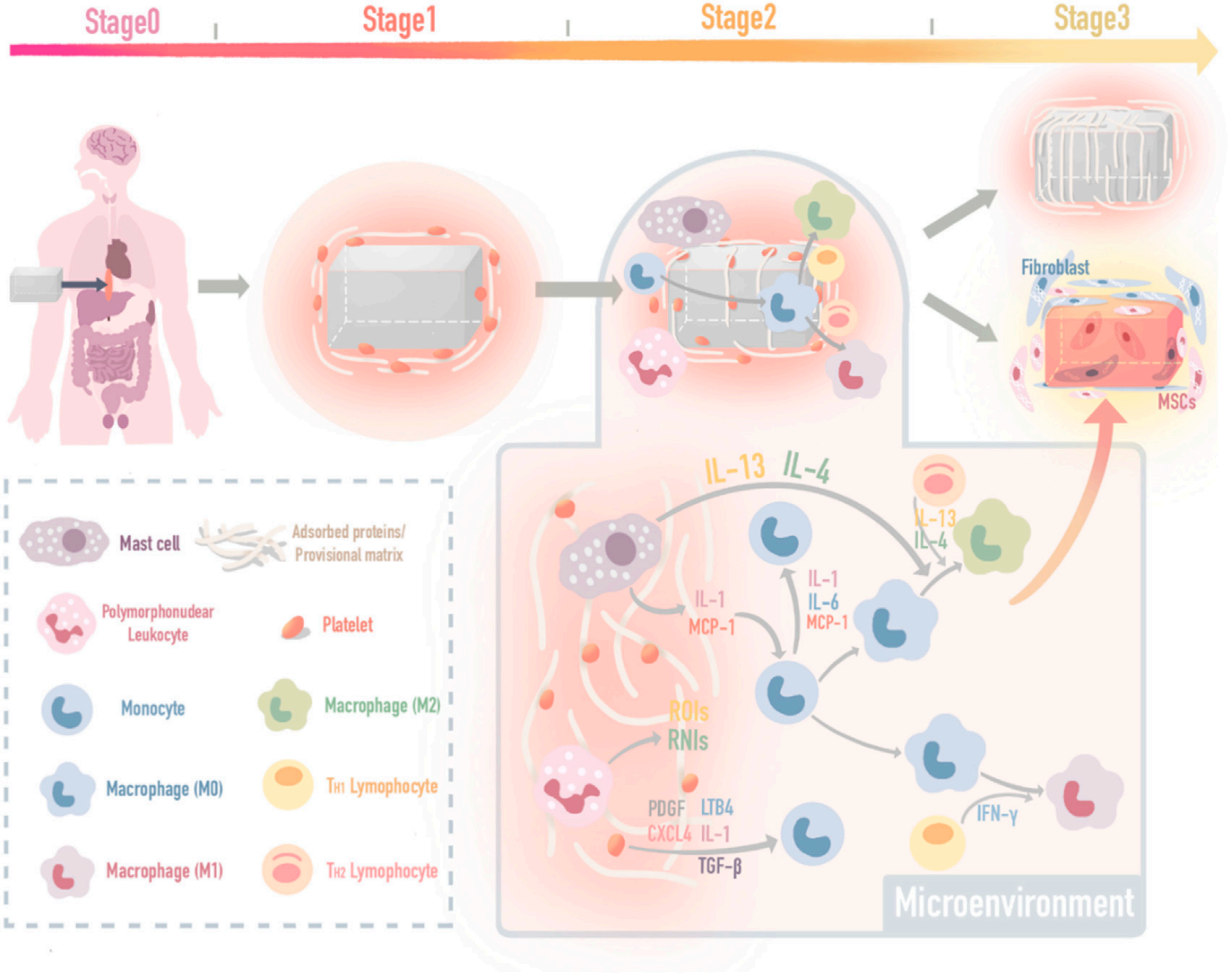
The mechanism map of three FBR stages. The mechanism and biological process of the foreign body response (FBR) induced by implants include three stages: protein adsorption, inflammation, and *in vivo* integration.

As several excellent reviews have elaborated on the mechanism and biological process of the FBR, we have briefly summarized these processes and divided them into the following three stages (Mariani et al., 2019; Gaharwar et al., 2020; Mukherjee et al., 2020; Zhang et al., 2021) (Figure 1)

1) **Protein adsorption.** The first FBR stage occurs within seconds, in which components of the blood, including fibrous protein immediately adsorbed onto the surface of the biomaterial and platelets adherent (forming a provisional matrix) (Barker and Engler, 2017; Wight, 2017; Mendes et al., 2018). The complement system in the host is activated at the same time (Barrington et al., 2001; Donat et al., 2019; Haapasalo and Meri, 2019), directly attacks the cells in the biomaterial implants and recruits neutrophil infiltration, resulting in vascular endothelial damage, fibrin deposition, and massive platelet aggregation around the implant (Park et al., 2018a; Rahman et al., 2018; Braune et al., 2019; Tanneberger et al., 2021).
2) **Acute inflammation.** The second stage occurs within a few hours to a few days. The provisional matrix contains many growth factors and chemokines, which recruit mast cells and multinucleated lymphocytes (Zhou and Groth, 2018; Lock et al., 2019; Teixeira et al., 2020). Mast cells release TNF-α, IL-1β, and MCP-1, which recruit monocytes and activate Toll-like receptors on the monocyte surface to stimulate maturity (Beghdadi et al., 2011; Maximiano et al., 2017; Komi et al., 2020; Ozpinar et al., 2021a). On this basis, TH1 lymphocytes (Th1) release IFN-γ to promote macrophage polarization toward the M1 phenotype (M1) (Mariani et al., 2019; Vassey et al., 2020), M1 macrophages release IL-1, IL-6, IL-8, IL-12, and TNF-α to mediate inflammation, and the protein mentioned above can further stimulate the polarization of macrophages (Wynn and Vannella, 2016; Zhou et al., 2020a; Engler et al., 2020).
3) **Host integration.** The third stage occurs a few days after the second stage, and its direction depends on the immunomodulatory results of the previous two stages. In the microenvironment with inflammation-related genes (IL-1β-related genes, etc.) upregulation (He et al., 2020; Nakkala et al., 2021a), FBR outcomes such as chronic inflammation, excessive granulation, collagen fiber deposition and fibrous tissue formation (Castaño et al., 2018; Adu-Berchie and Mooney, 2020; Gaharwar et al., 2020). In regard to FBR controlled, fibroblasts and mesenchymal stem cells (MSCs) are recruited to regenerate and continue a good repair process (Hannan et al., 2017; Soundararajan and Kannan, 2018; Wang et al., 2018).

## 3 Interaction Links: Cues for the Development of Immunomodulatory Biomaterials With Integrated Substances

**FIGURE 2:**
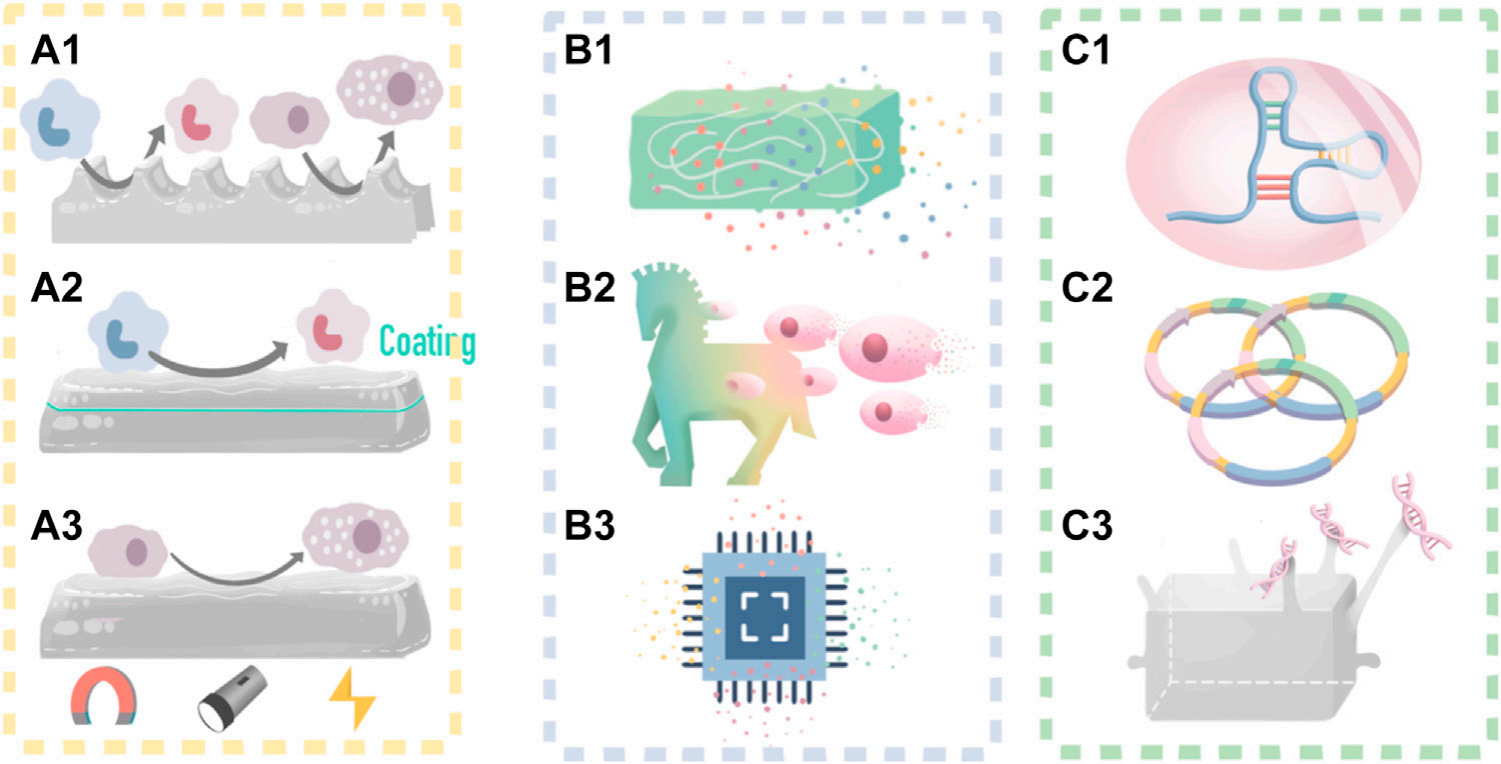
IMB decoration methods and a self-adaptive example. **(A)** IMB decoration with cues from cell; **(A1)** decorate surface morphology and mechanical properties to control macrophages and mast cells; **(A2)** add biochemical coating to control macrophage M2 polarization; **(A3)** control macrophage polarization by time-dependent change of external stimulus. **(B)** IMB decoration strategy with cues from protein delivery; **(B1)** decorate scaffold to be a delivery syetem; **(B2)** decorate MSCs to be a delivery syetem for high-targetting; **(B3)** integrated sustained-release chips. **(C)** IMB decoration with cues from nucleic acid; **(C1)** RNA interference; **(C2)** plasmid vectors; **(C3)** DNA grafting.

The FBR process remodels and integrates the implants into the immune microenvironment of the host by interacting with cells and bioactive substances. Therefore, the IMB should be designed with an “immune-informed” ability (Reid et al., 2018; Tang et al., 2018; Mariani et al., 2019; Adu-Berchie and Mooney, 2020; Zhang et al., 2021), precisely, the ability to regulate microenvironment bioactive substances to form feedback. In this way, the interaction between the FBR activity and IMB feedback can control the FBR by regulating bioactive substances. In this review, the interactions among regulating cells and bioactive substances which including proteins and nucleic acids are summarized, which provide cues for determining IMB incorporation strategies to achieve better tissue repair. (Figure 2).

## 4 Cell Interaction: Modifying Immunomodulatory Biomaterials to Modulate Immune Cell Functions

### 4.1 Macrophage Polarization

Macrophages play an important role in the second stage of the FBR (Wynn and Vannella, 2016; Petrosyan et al., 2017; Boada-Romero et al., 2020) and mainly exist as the pro-inflammatory M1 phenotype. The M1 phenotype secretes numerous matrix metalloproteinases (MMPs) and different cytokines, such as TNF-α, IL-1, IL-6, IL-8, and IL-10, which further stimulates the inflammatory response (Delavary et al., 2011; Vishwakarma et al., 2016; Wynn and Vannella, 2016; Zhang et al., 2016; Olingy et al., 2019; Davenport Huyer et al., 2020). Meanwhile, different macrophage phenotypes can arise in response to immune information (Sica and Bronte, 2007; Liu and Yang, 2013; Vassey et al., 2020; Muñoz-Rojas et al., 2021). Therefore, many researchers aim to design IMBs capable of transforming naive macrophages or M1 macrophages in the microenvironment into anti-inflammatory M2 macrophages and improve the anti-inflammatory ability and tissue repair function of the IMB (Sridharan et al., 2015; Wynn and Vannella, 2016; Ghasemi et al., 2019; Yin et al., 2020a; Zhou et al., 2020a; Engler et al., 2020). IMB modification methods can be focused on biophysical cues and biochemical cues.

#### 4.1.1 Biophysical Modifications

**FIGURE 3:**
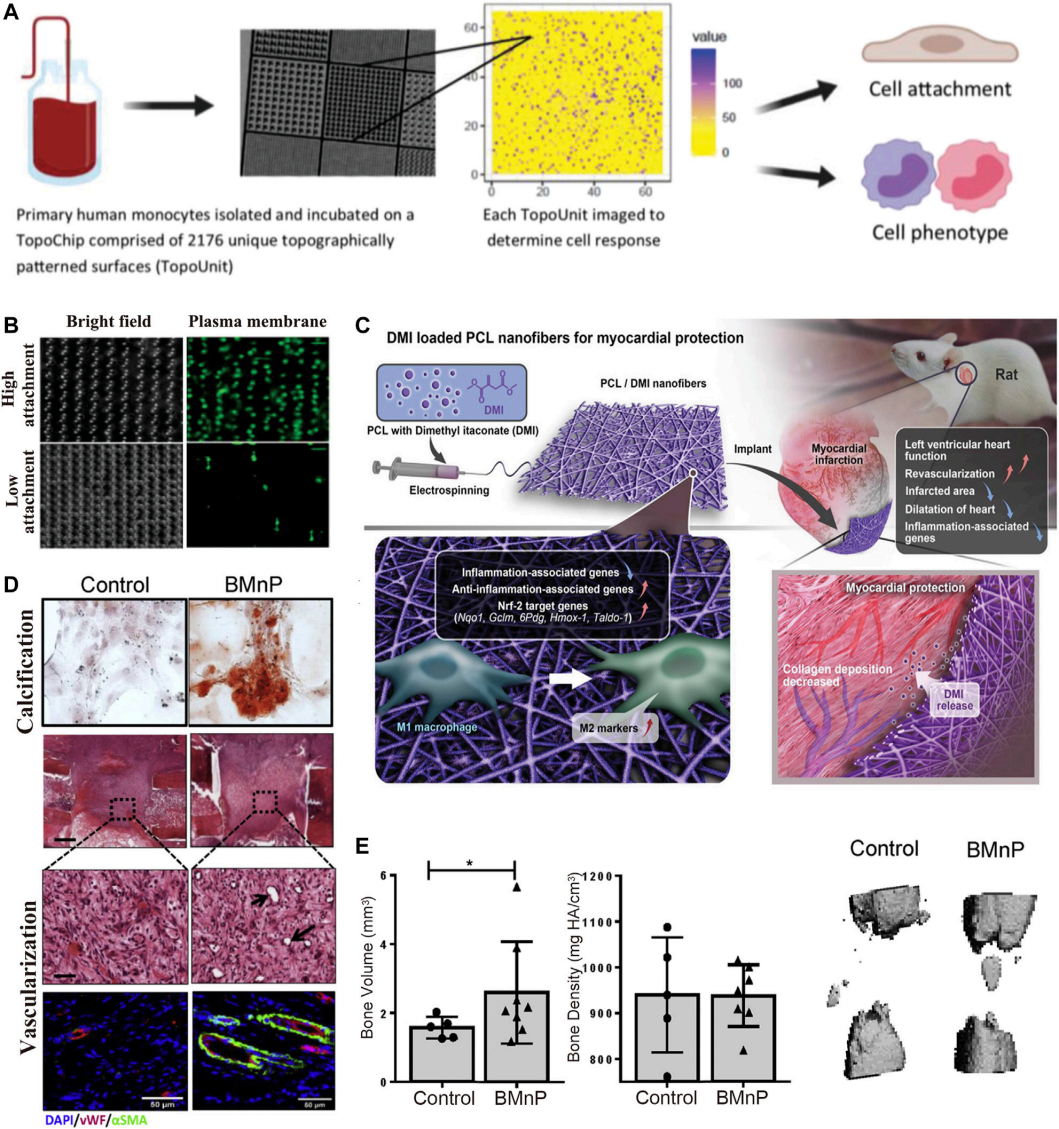
IMB Decoration with cues from macrophage polarization. **(A,B)** Reprinted with Creative Commons CC BY license(Vassey et al., 2020). **(A)** A high throughput screening approach is utilized to investigate the relationship between 2,176 micropatterns surface morphology and macrophage attachment and phenotype. **(B)** Micropillars 5–10 μm in diameter play a dominant role in driving macrophage attachment and M2 phenotype. **(C)** Dimethyl itaconate (DMI)-loaded PCL nanofibers and their roles on modulating the polarization of M1 into alternatively activated M2 macrophages, and protecting from myocardial infarction *in vivo* by improving left ventricular heart functions and down regulating inflammation-associated genes. Reprinted with permission from© 2022 WILEY (Nakkala et al., 2021b). **(D,E)** Nano-particle treated macrophages enhances osteogenic differentiation and vascularization. Reprinted with permission from© 2022 WILEY (Arizmendi et al., 2021).

Macrophage polarization can be controlled by biophysical cues, such as surface morphology (Sridharan et al., 2015; Zhou et al., 2020a; Davenport Huyer et al., 2020; Muñoz-Rojas et al., 2021) (Figure 2A1, Figures 3A,B).

In terms of mechanical properties, macrophages perceive the material’s rigidity through Rac-1 mechanosensory pathways, which then influence M1/M2 polarization (Acevedo and González-Billault, 2018; Guimarães et al., 2020; Healy et al., 2020). Many studies have demonstrated that M2 is the main direction of macrophage polarization on soft materials (Li et al., 2018; Guimarães et al., 2020; Atcha et al., 2021; Ye et al., 2021). For example, Blakney et al. (2012) have shown that when the internal rigidity of 3D polyethylene glycol-RGD is kept at 130 kPa (low stiffness), the proportion of M2 macrophages increases, upregulating the release of anti-inflammatory cytokines such as IL-10 and inhibiting the FBR. Yanlun Zhu et al. (Zhu et al., 2021) used rigid regulation cues and added bioactive glass to sodium alginate hydrogel to soften its mechanical properties, which effectively promoted M2 polarization and optimized the repair effect in damaged skin tissue.

Macrophages are sensitive to surface morphology changes larger than 5 μm, as a result of Rac-1 mechanosensory pathway and F-actin changes (Acevedo and González-Billault, 2018; Guimarães et al., 2020; Healy et al., 2020). Matthew et al. (Vassey et al., 2020) used a high-throughput method to screen the relationship between 2,176 types of surface morphology and macrophage attachment and phenotype. The results showed that modifying the IMB surface with microcolumns, retaining a diameter between five and 10 μm, yields excellent effects on macrophage attachment and M2 polarization.

Pore size is also an important driver of macrophage polarization. Fa-Ming Chen et al.(Yin et al., 2020b) proved that collagen-scaffolds with 360 μm sized pore promoted macrophages undergoing a higher degree of M1-to-M2 transition. Groll et al. (Tylek et al., 2020) pronounced that fibrous scaffolds with inter-fiber pore from 100 to 40 μm facilitated macrophage elongation accompanied by M2 polarization. Also, M2 polarization was reported in response to polyurethane scaffolds with 100 μm sized pore (Liang et al., 2018). In general, macrophages could undergo M2 polarization in macro pores with sizes ranging from tens of microns to hundreds of microns. The specific optimal pore size varies greatly among different materials due to the properties of scaffold materials, such as rigidity and elasticity.

#### 4.1.2 Biochemical Decorations

Chemical coatings and nanomaterial coatings are also feasible mainstream research directions (Davenport Huyer et al., 2020; Gao et al., 2020). For example, Nakkala et al. (2021b) showed that a dimethyl itaconate (DMI) coating on IMBs promoted the adhesion of M2 macrophage, and protected against myocardial infarction *in vivo* by improving left ventricular heart function. McBane and others studies have shown that coating IMBs hydrophobic ionic polyurethane (DPHI) has produces effective anti-inflammatory activities. Mahon et al. (2020) performed an immunomodulatory modification of bone defect healing biomaterials by adding nanohydroxyapatite particles (BMnP), which promoted M2 polarization, tissue angiogenesis, and increased bone mass (Figure 3).

### 4.2 Cues From Mast Cell Maturity

Mast cells also play an essential role in the second stage of the FBR (Beghdadi et al., 2011), in which mast cells are activated to a mature state by several receptors, such as FcɛRI, Toll-like receptor, and RIG-like receptor (Beghdadi et al., 2011; Komi et al., 2020). Mature mast cells release histamine, tryptase and monocyte chemoattractant protein-1 (MCP-1) activate fibroblasts, who in turn release stem cell factor (SCF) to continue regulating MCs through CD117, which promote beneficial tissue repair process, like neovascularization and so on (Maximiano et al., 2017; Ozpinar et al., 2021a). Therefore, the controller strategy can be applied on mast cells to accelerate mast cell maturity, and thereby more cytokines will be secreted. Additionally, modification methods can be focused on biophysical cues and biochemical cues.

#### 4.2.1 Biophysical Modifications

The maturation of mast cells can also be promoted by changing the material surface morphology (Atiakshin et al., 2018; De Zuani et al., 2018; Frossi et al., 2018; Chu et al., 2019; Sammarco et al., 2019; Galli et al., 2020; Ragipoglu et al., 2020; Yabut et al., 2020; Arizmendi et al., 2021). Maximiano et al. (2017) have indicated that, similar to macrophages, the maturity and functional activity of mast cells can also be controlled by the IMB surface morphology. For example, mast cells tend to adhere to and mature on IMBs constructed with large pore size microholes (Ozpinar et al., 2021b) (Figure 2A1).

#### 4.2.2 Biochemical Modifications

Maturation-associated receptors provide cues for accelerating the release of functional proteins from mast cells (Kempuraj et al., 2018; Olivera et al., 2018; Thangam et al., 2018; Widiapradja et al., 2019; Wilcock et al., 2019). Milmy et al. (Ortiz et al., 2020) demonstrated that PCL scaffolds modified to generate IMB scaffolds by the incorporation of dinitrophenyl IgE can activate FcɛRI to promote mast cell maturity and then further regulate the FBR and facilitate tissue repair by TNF-α and IL-13, which are released by mature mast cells. Modifications using that activate Toll-like receptors and c-type receptors are also a feasible method for “controlling” mast cell maturation and regulating the immune microenvironment (Ozpinar et al., 2021a).

### 4.3 More Promising Cues From Immune Cells

Macrophage polarization and mast cell maturation have been successfully promoted by IMBs, proving that modifying IMBs to “control” cell function is feasible. The highlight of this strategy is that immune information attached in a simple modification recognized by the cells in the microenvironment promotes the cells to differentiate as required for tissue repair. This strategy shifts the dominance of the interaction from the microenvironment to the material. The result of other studies have also indicated that other immune cells, such as T cells (Th1, Th2) (Choo et al., 2017; Wolf et al., 2019), dendritic cells (Eslami-Kaliji et al., 2020; Nguyen et al., 2020; Čolić et al., 2020), multinucleated lymphocytes, and even fibroblasts and MSCs (Soundara Rajan et al., 2020; Chang et al., 2021) in the microenvironment could be controllable by modified IMBs to further regulate the FBR.

## 5 Cues From Protein Interaction: Modifying Immunomodulatory Biomaterials to Optimize Protein Delivery

### 5.1 Modified Immunomodulatory Biomaterial as Protein Delivery Systems

Proteins, including interleukins, growth factors, and complement proteins, are bioactive constituents of the immune microenvironment, that interact with cells and nucleic acids to form a microenvironmental regulatory network. Therefore, protein delivery is a viable strategy to regulate the FBR. (Sharma et al., 2016). Determining which effector proteins should be selected and how to deliver these proteins is a leading research direction (Chung et al., 2017; Leach et al., 2019). (Table 1).

**TABLE 1:** IMB modulates FBR to meet better outcomes by delivering proteins.

Scaffold	Protein	Delivery mode	Outcome	References
PLGA/PDA/PCL composite scaffolds	Insulin	Single protein sustained release	The scaffolds stimulated chondrocytes proliferation, BMSCs differentiation, and enhanced bone and cartilage repair *in vivo*	Liming Wang Wei et al. (2021)
Nerve guidance conduit (NGC) scaffold	Melatonin (MLT)		NGC-MLT scaffold promoted morphological, functional, and electrophysiological recovery of regenerated sciatic nerves *in vivo*.	Wei-En Yuan Xu et al. (2020)
				Wei-En Yuan Chen et al. (2020a)
Tannic acid (TA) coating Ca-alginate scaffold	E7/P15 peptides	Synergistic sustained release of multiple protein	The scaffold induced BMSC recruitment and bi-lineage differentiation by E7 and P15, enhancing cartilage and subchondral bone regeneration	Jialin Chen Zhang et al. (2020)
Hyaluronic acid (HA) injectable scaffold	Stromal cell derived factor-1 (SDF-1); Kartogenin (KGN)		The regenerated tissue had the typical cartilage histological characters and integrated well with the surrounding tissue after 12 weeks of injection	Zhibing Zhang Wu et al. (2020)
A drug-releasing microporous annealed particle (drugMAP) system	Forskolin (F); Repsox (R)		FR/drugMAP treatment increased angiogenesis, reduced fibrosis and inflammatory response, and improved left ventricular functions	Song Li Fang et al. (2020)
Silk fibroin (SF)/nano-hydroxyapatite (nHAp) scaffold	Stromal cell derived factor-1 (SDF-1); Bone morphogenetic protein-2 (BMP-2)	A time-dependent sequential synergistic release of multiple protein	Scaffold increased bone regeneration in rat cranial defects, and the bone completely bridged the injury site after 12 weeks of implantation	Liang Chen Shen et al. (2016)
Porous mesoporous bioglass scaffold	Dexamethasone; Bone morphogenetic protein (BMP)		The scaffold regulated the recruitment and polarization of macrophage phenotypes and facilitated developmental bone growth process	Changsheng Liu Liu et al. (2021)
3D printed zinc oxide (ZnO) micro-particles hydrogel patch	vascular endothelial growth factor (VEGF)	Sequential release in spatiotemporal coordination started by stimulus (light, heat, magnetic, etc.)	The printed wound patches reduced immunogenicity and enhanced wound healing *in vivo*	Su Ryon Shin Siebert et al. (2021)
Integrating biomimetic 3D bioprinted fluid perfused microstructure	vascular endothelial growth factor (VEGF); Bone morphogenetic protein (BMP)		The microstructure benefited vascularized bone regeneration, improved complex vascularized tissue or organ regenerations	Lijie Grace Zhang Cui et al. (2016)

In terms of protein selection (Fisher et al., 2017; Frejd and Kim, 2017; Grim et al., 2018; Simeon and Chen, 2018; Mohammadinejad et al., 2019), Sharma and others have demonstrated that the interleukin family (IL) has effects several target cells (Akdis et al., 2016; Ghilardi et al., 2020). For example, IL-1 enhances immune function, IL-4 and IL-13 regulate the inflammatory response (Arend et al., 2008). Therefore, implants modified with these proteins can inhibit the FBR and are better integrated into the surrounding tissue. Meanwhile, growth factor (GF) is a polypeptide substance that regulates cell growth and its expression can be upregulated in an inflammatory microenvironment, which can promote vascular regeneration (Akdis et al., 2016; Zbinden et al., 2020). Vascular regeneration inhibits the FBR, and the implant can integrate in the host to facilitate tissue repair. Additionally, the complement protein family (Donat et al., 2019), including some oligopeptides (Zhang et al., 2020), has been considered for IMB modification, because it is a component of the innate immune system and plays a vital role in the first stage of the FBR (Barrington et al., 2001; Haapasalo and Meri, 2019), this stage of the FBR can destroy biomaterials directly and also recruit neutrophils to facilitate uncontrolled progression to later FBR stage (Panichi et al., 2000). These proteins are representative bioactive substances that have been widely investigated as IMB modifications.

In terms of the delivery system (Shadish et al., 2019; Takeuchi et al., 2017; Kuo et al., 2018; Rehmann et al., 2017; Leijten et al., 2017), (Figure 2B), most biomaterials, such as alginate, PEGate-gelatin scaffolds, and collagen/hyaluronic acid scaffolds, possess their own slowly releasing proteins for internal charge adhesion and porosity, indicating that it is feasible to attach a protein delivery system to IMBs. One promising example is the use of a polyelectrolyte multilayer coating (PLG-scaffold) (Deng et al., 2020) to enhance the sustained-release function of IMBs, as the thickness of the coating can be easily modified to achieve different hydrophilic protein levels and release rates. For example, David et al. (Li et al., 2020) used a PLG coating modification for the sustained-release of steroid drugs to reduce aseptic inflammation in nerve prosthesis transplantation. Additionally, IMBs can be modified to carry multiple proteins through direct protein mixing and the use of integrated chips (Sharma et al., 2021). On this basis, a strategy for the sequential release of proteins in spatiotemporal coordination initiated by an external stimulus [light, heat (Gnaim and Shabat, 2019), magnetic (Orapiriyakul et al., 2020), acoustic wave (Moncion et al., 2017), etc.] has been proposed to align the IMB function with the tissue repair process in the body (Jimi et al., 2020; Oliva and Almquist, 2020) (Figure 2B3).

### 5.2 Optimal Immunomodulatory Biomaterial Modification: Highly Targeted Delivery Systems

To improve targeted delivery, many researchers have modified stem cells (Zhou et al., 2020b; Su et al., 2020), T cells (Choo et al., 2017; Cevaal et al., 2021), biological vesicles (Anika Nagelkerke, 2020; Brennan et al., 2020), and other vehicles into delivery systems due to their excellent biocompatibility and targeting ability to eliminate the problem of proteins diffusing locally around the implants (Jin et al., 2018).

For example, the Martinez team creatively proposed combining MSCs with nanocarriers to construct a multifunctional multicomponent “M&M delivery platform”. This IMB platform takes advantage of the inflammation-targeted function of MSCs, and the drug is accurately targeted and delivered to the activated immune microenvironment. IMBs constructed with a combination of a targeted carrier and a bioactive substance are vividly defined as “Trojan horses” (Martinez et al., 2021) (Figure 2B2). Wang et al. (Wang et al., 2017) demonstrated the feasibility of these IMBs by coating bioactive substances with platelet extension vesicles (PEVs) to regulate bleeding and protein deposition in the FBR.

The protein delivery strategy is the most widely used modification strategy at present (Ooi et al., 2017; Rosales et al., 2017; Brown, 2018; Hedegaard et al., 2018; Jain et al., 2018); the biomaterial itself is a suitable carrier for many regulatory proteins, and extensive knowledge of cytokines (interleukin family, growth factors, chemokines, etc.) in immunology provides a foundation for its application. The highlight of this approach is that in the material-microenvironment interaction process, the proteins are not only involved in cell and nucleic acid interactions but are also the main component of the FBR.

Therefore, the protein delivery strategy is simple and effective, and IMBs carrying proteins can directly prevent the progression of the FBR. Moreover, the targeted optimization of the “Trojan Horse” approach allows the delivered protein to act more specifically in the immune microenvironment, improves the efficacy of tissue repair and reduces the potential for a systemic response.

## 6 Cues From Nucleic Acid Interactions: Modifying Immunomodulatory Biomaterials Using Genome-Editing Techniques

### 6.1 Modifying Immunomodulatory Biomaterials to Determine Cell Fate and to Form a Regenerative Microenvironment

Recently, with the development of molecular biology and genome-editing techniques, a deep molecular understanding of the FBR has been obtained, which has provided cues for applying genome-editing techniques for the modification of IMBs (Mali and Cheng, 2012; Nelson and Gersbach, 2016; Kamburova et al., 2017; Glass et al., 2018; Metje-Sprink et al., 2019; Mushtaq et al., 2019; Vats et al., 2019; Ali et al., 2020). This approach can be used to generate IMBs that can regulate the immune cells, stem cells and fibroblasts recruited in the third stage of the FBR (Hiew et al., 2018; Ma et al., 2018; Ng et al., 2021). After using genome-editing techniques to integrate nucleic acid information into repair-related cells, such as MSCs, repair-related proteins such as growth factors are secreted, which promote a microenvironment more conducive to tissue repair. Additionally, the nucleic acids transcribed by MSCs can directly regulate stem cell fate, determine the direction of differentiation of specific cell types, and complete the repair of specific structures.

It is novel to use nucleic acids as an upstream regulation strategy. The progression of the FBR and the characteristics of the microenvironment are influenced by IMBs modified in this manner. This modification method targets earlier in the repair process than cellular- and protein-level modifications and is widely applicable for tissue repair and other directions. Of course, more research is still needed to determine the safety, ethical requirements, and stability of this approach.

### 6.2 Examples of Genome-Editing Techniques Applied for Immunomodulatory Biomaterial Modification

#### 6.2.1 The RNA Interference (RNAi) Technique

**FIGURE 4:**
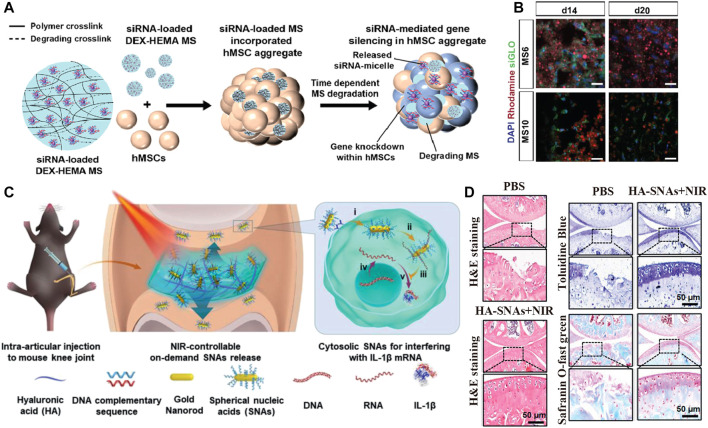
IMB Decoration with cues from nucleic acid-associated technologies. **(A,B)** Reprinted with permission from Copyright© 2022, Elsevier (Alexandra McMillan et al., 2021). **(A)** Schematic show the fabrication of DEX based MS encapsulating siRNA-micelles, and depicte the incorporation of siRNA-MS into a MSCs aggregate for localized and sustained siRNA presentation and subsequent sustained gene silencing within a stem cell aggregate. **(B)** Distribution of incorporated MS in MSCs aggregates for sustained siRNA presentation. Fluorescence confocal photomicrographs of rhodamine-labeled (red) siGLO-MS incorporated into MSCs aggregates to visualize siGLO uptake (green) and DAPI stained MSCs nuclei (blue) in 3D aggregates after different culture periods **(C,D)** Reprinted with Creative Commons CC BY license (Chen et al., 2020b). **(C)** NIR light-controllable SNAs release based on DNA-grafted HA for OA treatment. HA-SNAs system is injected into the knee joint and irradiated by NIR light to gradually release the SNAs, which enter into cells to interfere with mRNA molecules to silence IL-1 expression. **(D)** Histological staining shows the treatment of mouse after injection of PBS and HA-SNAs + NIR at 12 weeks after surgery.

Alexandra McMillan et al. (2021) encapsulated RNA nanocomplexes in IMBs to construct IMBs with RNA interference (RNAi) technology to influence the cell fate decision at the messenger RNA (mRNA) level. The properties of IMB ensure the long-term retention and effectiveness of RNA nanocomplexes *in vivo*. The results proved that RNA nanocomplexes were still locally functional after 28 days and controlled the fate of stem cells, which differentiated into osteoblasts and chondrocytes, providing a new strategy for bone repair (Figure 2 C1, Figures 4A,B).

#### 6.2.2 The Plasmid Transfection Technique

In the study of Moreira (Moreira et al., 2021), plasmid vectors were attached to IMBs for the continuous production of repair- and regeneration-related proteins (e.g., VEGF and FGF) to promote tissue regeneration and repair. In this study, based on the original porous sponge material, chitosan (Ch) and polyethyleneimine (PEI) were used as nonviral vectors to transfer the plasmid encoding vascular endothelial growth factor (p-VEGF) and the plasmid encoding fibroblast growth factor-2 (p-FGF-2). The results showed that plasmid DNA rapidly produced these growth factors in the microenvironment, which induced the formation of capillary-like structures and promoted the assembly of endothelial cells into several capillary segments (Figure 2C2).

#### 6.2.3 The DNA Grafting Technique

Chen et al. (2020b) modified hydrogel scaffolds by spherical nucleic acid and DNA grafting to regulate the immune microenvironment. Chen grafted complementary strand DNA onto hyaluronic acid to obtain ^DNA^HA and then combined spherical nucleic acids (SNAs) by base pairing to form an SNA-^DNA^HA system. The DNA was unhybridized by photothermal induction, and the SNAs were released to downregulate the expression of inflammation-related genes, such as the IL-1β gene and the protease MMP gene, and upregulate the expression of matrix synthesis genes, such as the collagen II gene, thus controlling the inflammation caused by the FBR (Figure 2C3, Figures 4C,D).

## 7 Cues From the Spatial-Temporal Heterogeneity of the FBR: Optimal Immunomodulatory Biomaterial Modifications for Responsiveness

### 7.1 Spatial-Temporal Heterogeneity of the FBR: Requirement for Responsive Immunomodulatory Biomaterials

As described above, the FBR involves the integration of biomaterials into the immune microenvironment of the host through interaction with bioactive substances, such as cells, proteins, and nucleic acids. These interactions provide cues for biomaterial modification strategies, which allow IMBs to modulate the FBR and induce the evolution of the FBR toward tissue regeneration and repair (Campi et al., 2017; Lalitha Sridhar et al., 2017; Rose and De Laporte, 2018; Ashammakhi et al., 2019; Gonzalez-Fernandez et al., 2019; Riley et al., 2019; Daly et al., 2021).

However, the FBR and tissue repair occur in stages and have spatial-temporal heterogeneity; that is, the various bioactive substances form divergent interactions at different temporal points. The concept of responsive IMBs was proposed for modulating multiple interactions, in which the IMBs can respond to environmental or external stimuli and provide diverse immune information feedback at different stages of the FBR. In other words, responsive IMBs exert a combination of the effects of the three IMB modification strategies mentioned previously, and can produce the effect of cascade amplification in tissue repair.

Responses to both external and environmental stimuli have individual advantages, and both are promising targets for the development of responsive IMBs. External stimuli are artificially imposed and can be better controlled. Additionally, the design of biomarkers responsive to external biomaterials is achievable. Environmental stimuli arise from changes in the immune microenvironment, and the temporal point of the response is more reasonable, but it is uncontrollable *in vivo*, thus requires more rigorous design.

### 7.2 Utilizing External Stimuli to Form a Multiple-Stage Regulation

If the original three-dimensional material structure is endowed with the ability to respond to external stimuli, such as heat, light, magnetic, and so on, the modified IMBs can exert different functions over time, which allows for diverse regulation.

**FIGURE 5:**
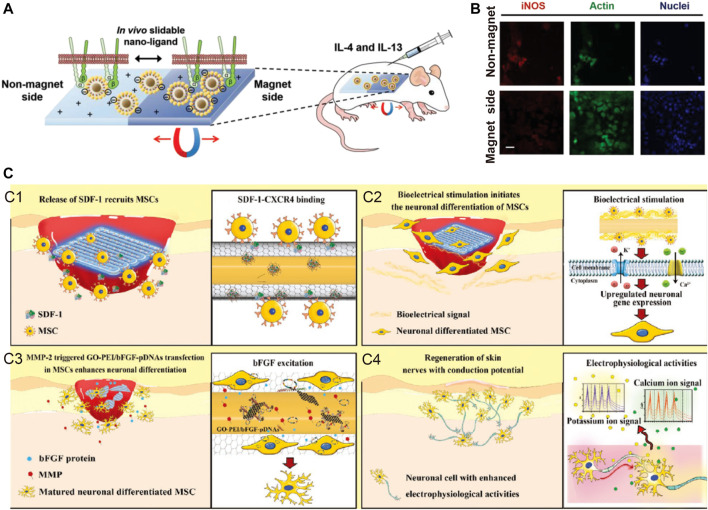
Examples of external stimuli and environmental stimuli. **(A,B)** Time-dependent magnetic attraction of the slidable nano-ligand facilitates macrophages adhesion, and stimulate regenerative M2 phenotype. Reprinted with permission from© 2022 WILEY (Choi et al., 2020). **(C)** Self-adaptive chip complete the damage skin repair with neuronal function by stimulating the nerve fiber formation and excitation function recovery with four major steps. Reprinted with permission from© 2022 WILEY (Li et al., 2020).

For example, magnetic nanomaterials are feasible carriers for the implementation of this strategy, and Choi and others have indicated that magnetic control of nanoligands can promote tissue regeneration (Choi et al., 2020). In their study, time-dependent magnetic stimulation was used to promote nanoligands carrying integrin-binding ligands (such as RGD) to aggregate in one area. Thus, the ligand density can be increased at a certain time point. When dense ligands aggregate the macrophage adhesion structure and promote the elongated assembly of actin, M1 phenotype polarization is inhibited, and M2 polarization is promoted. There are other examples of the use of external stimuli, such as ultraviolet (UV) light, which is used to trigger UV-mediated photolysis molecules and form different surface morphologies at different times, and this method has also been shown to be feasible for modulating various immune cell functions. (Figures 5A,B).

### 7.3 Utilizing Environmental Stimuli to Form a Self-Adaptive Regulation

Self-adaptive regulation of IMBs can provide diverse immune information feedback after initiation by changes in the immune environment, such as photothermal changes, pH changes, changes in the metabolites in the microenvironment, and so on, to inhibit multistage FBR and promote tissue repair.

Cui et al. (Cheng et al., 2020b) exploited the double responsiveness of NIPAAm molecules to explore this approach. When the FBR causes inflammation, the accumulated metabolites change the pH of the microenvironment and exceed the response threshold of NIPAAms, and NIPAAms respond to pH changes by releasing proteins through “gel transformation”, thus using the immune microenvironment as a method to activate the IMBs.

Gao et al. (Peng et al., 2020) designed a self-adaptive skin repair IMB, further demonstrating the feasibility of this approach. Self-adaptive IMBs can first increase the recruitment of MSCs into the microenvironment with a protein release strategy, then respond to the accumulation of stem cell matrix metalloproteinase (MMP) and release pFGF/DNA, promoting the neuronal differentiation of MSCs through pFGF/DNA, and ultimately, lead to the repair and functional recovery of damaged skin. Zhang et al. also used MMP to degrade the outer scaffold and to realize the sequential release of VEGF and BMP in temporal coordination and produce better bone repair, as VEGF-induced vascularization provides a foundation for vascularized bone regeneration.

## 8 Discussion: Intelligent Immunomodulatory Biomaterials

Self-adaptive responses can initiate multiple superimposed interactions at the level of cells, proteins and nucleic acids, provide various stimuli required for regulating the FBR and repair processes, and produce cascading amplification effects. Meanwhile, IMBs with high targeting capacity are required for specificity, and the previously mentioned “Trojan Horse” approach has been developed to achieve this. Therefore, developing IMBs that provide self-adaptative feedback over time and have high targeting capacity within specific areas is a future direction of IMB modification research (Veiseh et al., 2015; Tzu-Chieh et al., 2021).

Nowadays, IMBs have promising application prospects, but there is still a gap between clinical uses. In the future, while the design of IMB is being optimized, biocompatibility and biosafety evaluation should also be necessary to demonstrate the safety of the final product. Meanwhile, cadaveric and clinical studies should be performed to validate that the product’s safety and efficacy could meet preset clinical needs.

In addition, for modifications involving cells and bioactive substances which including proteins and nucleic acids, high throughput screening can be used as a reference to determine targets (Park et al., 2018b; Seo et al., 2018) and for more advanced mathematical modeling and big data analysis methods (Yang et al., 2021), which may result in better outcomes for screening surface morphology, modeling interactions between cytokines, and so on. Additionally, this approach will improve the stability and effect of single interactions and provide a foundation for intelligent self-adaptive tissue repair.
